# A novel heterogeneous acid–base nano-catalyst designed based on graphene oxide for synthesis of spiro-indoline-pyranochromene derivatives

**DOI:** 10.1186/s13065-023-00930-5

**Published:** 2023-03-10

**Authors:** Soghra Khabnadideh, Khashayar Khorshidi, Leila Amiri-Zirtol

**Affiliations:** Pharmaceutical Science Research Center, Shiraz University of Medical Sciences, Shiraz, Iran

**Keywords:** Graphene oxide, Organocatalyst, 3-Aminopyridine

## Abstract

Nano graphene oxide/3-aminopyridine has been introduced as a new, efficient and robust heterogeneous organic catalyst for synthesis of spiro-indoline-pyranochromene derivatives. Nano graphene oxide/3-aminopyridine was provided in an easy and green way from GO. Firstly, graphene oxide (GO) was synthesized and then 3-aminopyridine was immobilized with covalent bonds on its surface as a nitrogenous organic compound, in this step we didn’t use any organic or toxic substance. This bonding was easily performed due to the presence and reactivity of the epoxy groups in the GO structure. Because of its vast-surface nano-layers, GO could be effective in appropriate dispersion of 3-aminopyridine on its surface and increasing the catalyst performance. The new catalyst was analysed using different microscopic and spectroscopic techniques such as Fourier-transform infrared (FT-IR), field emission scanning electron microscope (SEM), energy-dispersive X-ray spectroscopy (EDS), X-ray diffraction (XRD) and thermogravimetric analysis (TGA). Our results showed that the distance between GO plates was increased in the presence of the modifying agent. This is due to the placement of the organic compound between the GO sheets. Finally, the ability of our new nano-catalyst in the synthesis of some spiro-indoline-pyranochromene and dihydropyranochromene derivatives was evaluated and acceptable results were obtained. Eight analogous of spiro-indoline-pyranochromene ***(4a-4 h)*** were synthesized in high yields and characterized. Using 3-aminopyridine as an organic and efficient catalyst, its stabilization by a simple method on GO, recycling of the catalyst up to 7 times and obtaining a highly pure product were the points that made the present work more attractive.

**Graphical Abstract:**

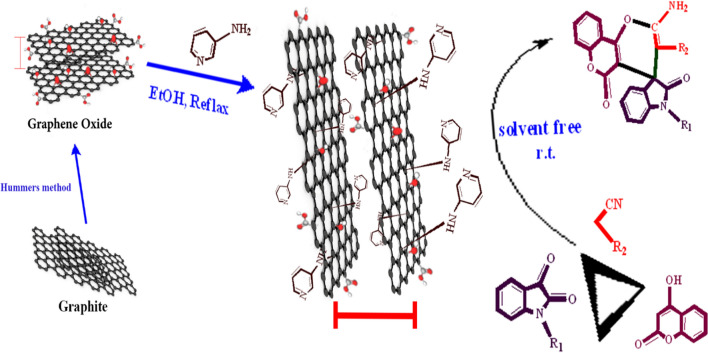

**Supplementary Information:**

The online version contains supplementary material available at 10.1186/s13065-023-00930-5.

## Introduction

Organocatalysts are now attracted many researchers due to their catalytic properties. They can be successfully implemented as a strategy for greener synthesis of various chemical and pharmaceutical compounds. These catalysts have the advantages of: cheapness, availability, efficiency and sufficient stability for reuse. Absence of metals in their structures and therefore, elimination of their contamination and high economic benefits are the other considerable advantages of organocatalysts. Being easily recovered and reused in organic reactions makes these catalysts also economically and environmentally desirable [1–5].

Different kinds of supports are prevalently used to improve the quality of these catalysts [6–8]. Among these, natural-based compounds (especially carbon-based) have received the most attention due to their consistency with green chemistry and their diverse structure. Vast layered structure, different functionalizing groups, lower intrinsic mass and resistivity against chemical changes are some of the advantages of GO as a carbon-based material [9–11]. The specific structure of GO allows it to interact with a wide range of organic molecules through covalent or non-covalent bonds (hydrogen bonds, electrostatic forces, Vander Waals forces, and hydrophobic interactions) [12–14].

The unique structure of some small organic molecules attributes in their exploitation as an orgnocatalyst. 3-Amino pyridine, for instance, with an amino group attached to pyridine ring acts as a basic catalyst because of the presence of two nitrogen’s with free electrons [15, 16].

Panahi et al*.* designed and synthesized a new heterogeneous catalyst using n-methyl-4-pyridinamine and GO. In their study, binding of the organic compounds on the edge of GO was achieved by thionyl chloride which is a toxic and unsafe substance. Thionyl chloride converted the carboxylic acid groups of GO to the active chloric acid groups in a harsh condition. n-Methyl-4-aminopyridine was then stabilized by attacking these groups on the GO wall. Some of the disadvantages of their method are prolonged reaction time and difficult synthesis of the catalyst [17]. Mirjalili et al*.* also designed a heterogeneous organocatalyst by placing 1,5-diazabicyclo [4.3.0] non-5-en on a SiO_2_ substrate. Using of volatile and hazardous materials such as n-hexane and thionyl chloride as well as the difficulty and costly path of the catalyst synthesis were among the weaknesses of this study [18].

On the other hand, multi-component reactions are suitable methods for the synthesis of chemical and pharmaceutical compounds. Spiro-indoline-pyranochromene derivatives are also synthesized through a multi-component reaction. These compounds showed a variety of biological properties due to the presence of isatin in their skeleton and are therefore of interest to many researchers. Synthesis of several derivatives of these compounds has been investigated so far. Many methods have been introduced including: using of triethylamine, polyoxometalate [H_2_ [MIMBS]_4_ [P_2_W_18_O_62_]0.11H_2_O] (MIMB-P_2_W_18_)_,_ and Na_2_EDTA, Fe_3_O_4_@l-arginine as catalysts [19–22]. Expensive catalysts, organic and toxic solvents and difficult separation of the product could be count as the disadvantages of the previous studies.

**Fig.1:**
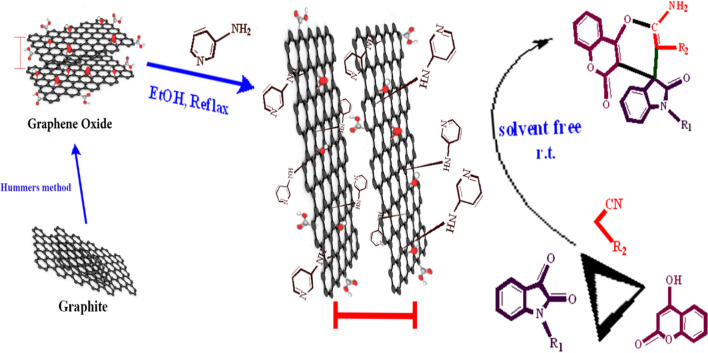
Graphical abstract

In the present work, paying attention to GO from special viewpoints has led to easy manufacture of a new catalyst without any toxic substances for fixing of 3-aminopyridine on the GO surface. In this study, a new heterogeneous nano catalyst with dual acid–base properties is introduced by stabilizing of 3-aminopyridine on the surface of GO (Fig. 1). Using of GO in this work has several advantages such as having a wide area, existence of numerous groups and low inherent mass. No toxic or volatile substances are employed in the second step because of the presence of epoxide groups, and the catalyst is prepared in a mild condition. Placement of 3-aminopyridine on the surface of GO sheets increases the distance between the sheets, and as a result, it facilitates easier access to these molecules and improves catalyst performance. Consistent with green chemistry, convenient and inexpensive methods for catalyst synthesis are notable points of the present study. In the created catalyst, the free N of the pyridine ring and the presence of an acidic part in graphene oxide create a catalyst with acid–base properties suitable for the reaction. The new catalyst was then characterized by different spectroscopic methods, and its efficiency for synthesis of spiro-indoline-pyranochromene and dihydropyranochromene derivatives was evaluated. The target compounds were synthesized in mild conditions, in a short time and good yields in the presence of the new catalyst. From the above category of compounds eight analogues of spiro-indoline-pyranochromenes **(4a-4h)** were prepared and characterized. Finally, the reusability of the catalyst was investigated as well.

## Materials and methods

All chemicals and solvents were prepared purely from Merck & Aldrich. Reaction progress was examined by thin-layer chromatography on PolyGram SILG / UV254 plates. Melting points of the synthesized compounds were measured by a Buchi B-540 B device. FT-IR analysis was performed to identify the synthesized compounds by Bruker. The reported FT-IR spectra were taken by KBr tablets in the range of 400 to 4000. Proton and carbon nuclear magnetic resonance (^1^HNMR, ^13^CNMR) spectra were recorded in Bruker (DRX-400 Avance) and DMSO-d_6_ was used as the solvent. FE-SEM (MIRA3TESCAN-XMU) was used to evaluate and compare the surface of the composite and GO. SAMX MIRA II was used for EDS analysis. Composite crystallographic characterization was performed by X'Pert PRO MPD P decomposition using Ni-FILTERED filtered Cu-K rays in the diffraction angle range of 5–80. Thermal decomposition analysis was performed by STA 505 under argon atmosphere.

## Chemistry

### Synthesis of nano-GO/3-aminopyridine

**Fig. 2:**
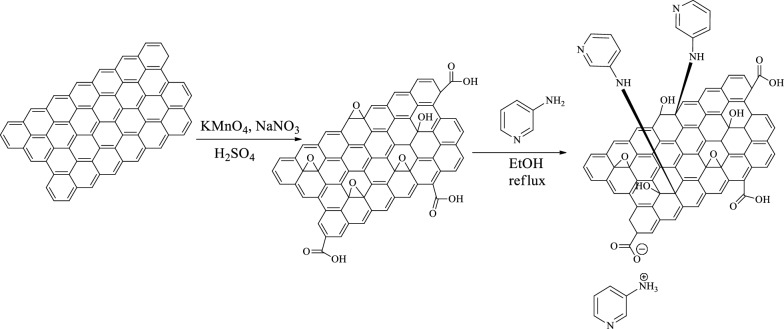
Synthesis of nano-GO/3-aminopyridine

Nano-GO/3-aminopyridine was synthesized in two steps. GO was first prepared according to the literature [23]. Then 0.5 g of GO powder in 20 mL of ethanol was well dispersed for half an hour in the bath sonication (KQ100DB ultrasonic cleaner, Kunshan, China, 100 W, 40 kHz). then 3-aminopyridine (0.5 g) was added to the reaction vessel and then allowed to stir for 12 h at reflux temperature. Finally, the obtained catalyst was centrifuged, washed with hot ethanol and dried overnight at 60 °C for 24 h (Fig. 2).

### Synthesis of spiro-indoline-pyranochromene derivatives

**Fig. 3:**
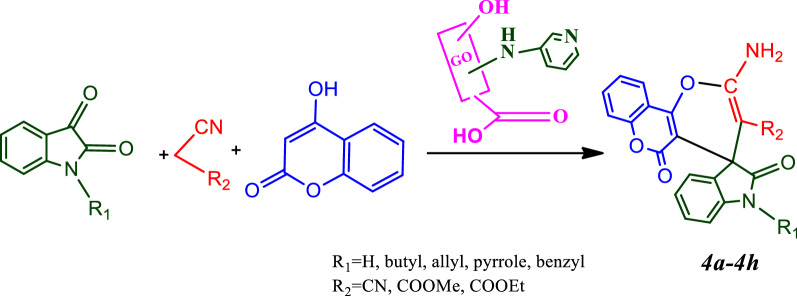
Synthesis of spiro-indoline-pyranochromene derivatives

N-substituted isatins were synthesized by the process expressed in the reported articles [19]. A mixture of N-alkyl or N-aryl isatin (1.0 mmol), nitrile moiety (1.0 mmol), 4-hydroxy coumarin (0.162 g, 1 mmol) and nano-GO/3-aminopyridine (0.4 g) was poured into a round bottom flask and stirred under solvent-free conditions at room temperature. After completion the reaction (monitored by TLC) the catalyst was separated by centrifugation. The resulting product was crystallized in ethanol for further purification to give the final product (Fig. 3).

## Results and discussion

Nano-GO/3-aminopyridine was easily synthesized in two steps under mild conditions. First, GO was synthesized according to the Hummer method [24]. 3-Aminopyridine was then fixed on GO sheets in ethanol at reflux temperature. The presence of epoxy rings in GO makes it easy to bind to the organic molecules via covalent bonds without any need to the toxic activators. Opening of these rings with nitrogen atoms of 3-aminopyridine confirms the successful fabrication of the catalyst. GO, with its large surface area, contributes to the proper dispersion of 3-aminopyridine in the catalyst structure, which leads to the use of the right amount of catalyst in the reaction. Placement of 3-aminopyridine on the surface of GO sheets increases the distance between the sheets and thus increases easier access to these molecules and results in better catalyst performance. Go with a wide surface area is a suitable support for organocatalysts. The basic component of the catalyst increases the effective reaction of the basic groups to the starting materials and subsequently the catalytic activity also improves. The presence of free nitrogen atoms in the pyridine ring and the presence of acidic GO fragments in the structure of this catalyst creates an acid–base catalytic property that is suitable for most chemical reactions. The chemical structure of the catalyst was investigated using FT-IR, XRD, FE-SEM, and TGA techniques. Then its catalytic activity in the synthesis of spiro-indoline-pyranochromene compounds was evaluated. Some spiro-indoline-pyranochromene derivatives were synthesized and their purity was confirmed by comparing characteristics with the literature reports.

### Catalyst identification

#### FT-IR spectra of nano-GO/3-aminopyridine

**Fig. 4:**
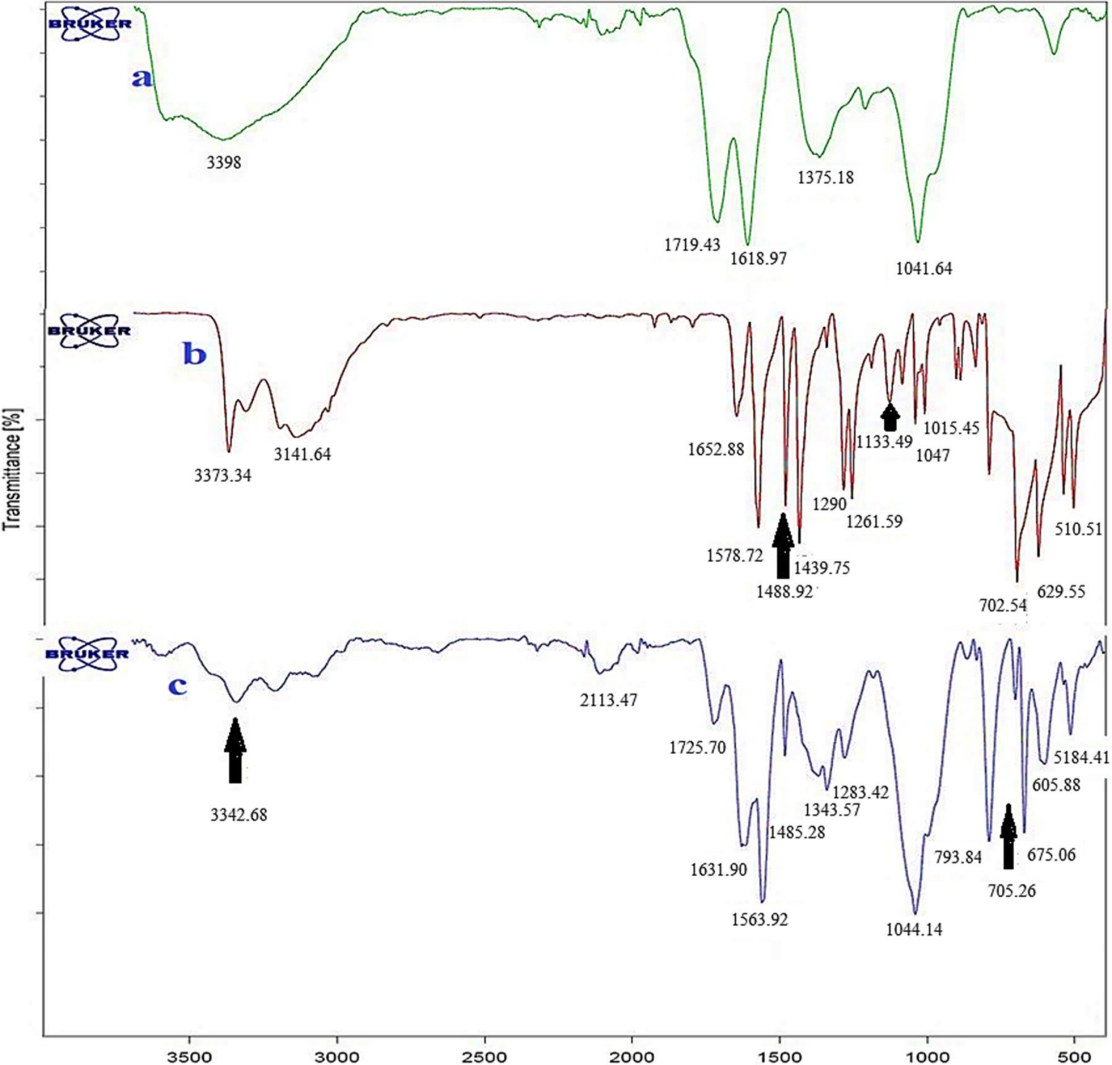
FT-IR spectra of GO (**a**), 3-aminopyridine (**b**), and nano-GO/3-aminopyridine (**c**)

The FT-IR spectra of GO, 3-aminopyridine, and nano-GO/3-aminopyridine are shown in Fig. 4. By comparing the spectra of GO and GO/3-aminopyridine, the interaction of absorption bands in GO before and after functionalization can be investigated. In the FT-IR spectrum of GO, the absorption peak at 3398 cm^−1^ is related to the stretching vibrations of the OH groups on the GO sheets and carboxylic acid groups in the GO wall. Two peaks in the regions of 1719 cm^−1^ and 11,618 cm^−1^ related to the stretching vibrations of the C=O and C=C groups and the peaks related to C–O in the area of 1041 cm^−1^ could be observed [25].

There are also significant peaks in the 3-aminopyridine spectrum. The peaks in the area of 3373 cm^−1^ and 3316 cm^−1^ belong to the NH_2_ stretching group. Absorption in the region of 3342 cm^−1^ is related to the stretching vibration of the NH group. The adsorption peak in 3199 cm^−1^ is related to C–H. The adsorptions in the area of 1652 cm^−1^ and 1486 cm^−1^ are related to the aromatic ring [15].

The FT-IR spectrum of the functionalized GO has quite pronounced changes. The addition and removal of some peaks indicate the placement of organic molecule on the surface of GO.

#### FE-SEM image of nano-GO/3-aminopyridine composite

**Fig. 5:**
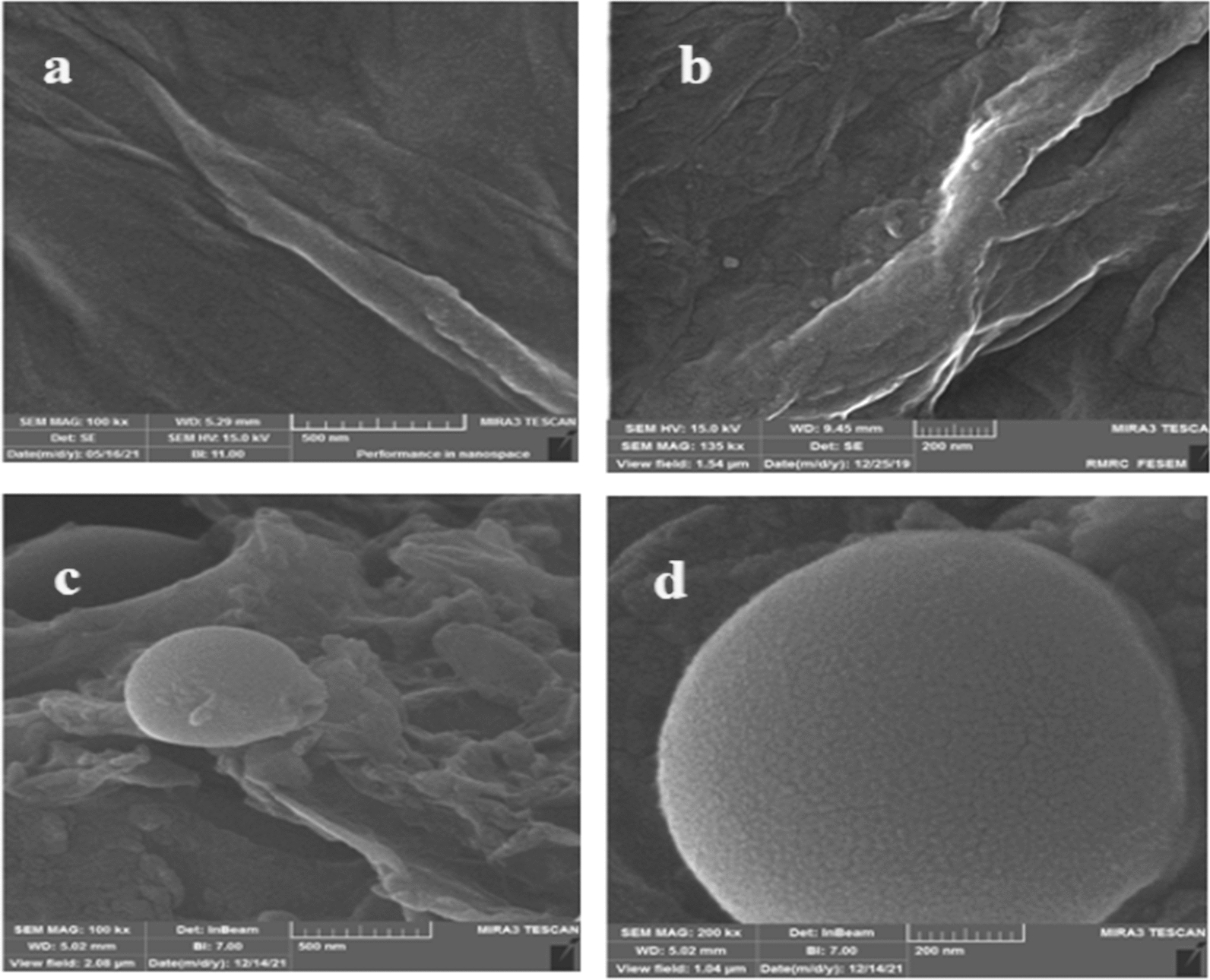
SEM image of GO (**a**–**b**) and nano-GO/3-aminopyridine (**c**–**d**)

The morphology of GO and nano-GO/3-aminopyridine are compared in Fig. 5. In the image taken from the GO surface (Fig. 5a, b), irregularities and wrinkles in the structure can be easily seen. Figure 5c, d shows a picture of the functionalized GO surface, which has visible changes on its surface that can confirm the functionalization of GO. The FE-SEM image of the functionalized GO surface confirms the presence of multiple grains on the surface and the morphological changes. As Fig. 5 shows, the layer thickness and morphology of GO sheets were completely changed after placing small molecules on the surface of GO. The highlighted particles on the catalyst surface are evidences for these changes on the GO layers. Actually SEM analysis shows only particle size and morphology of the surface area of GO.

#### Surface elemental analysis

**Fig. 6:**
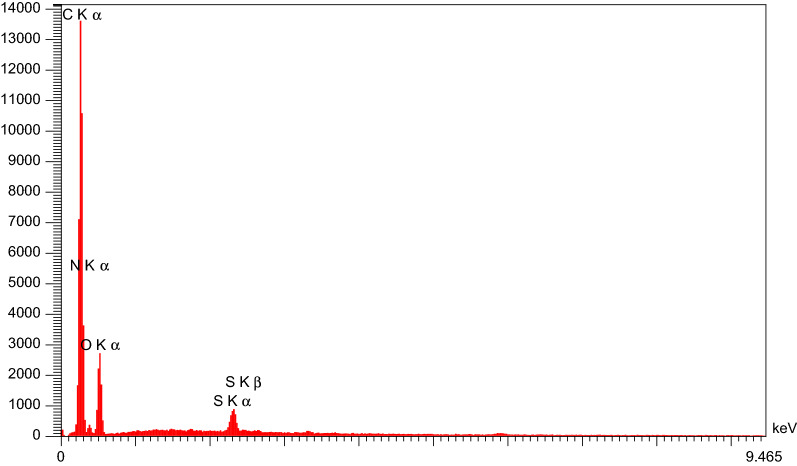
EDS analysis of nano-GO/3-aminopyridine

The EDS analysis of the composite in Fig. 6 shows the presence of the N element in the catalyst structure in addition to the O and C elements in GO. This result confirms the presence of 3-aminopyridine in the composite structure. Using this analysis, the percentages of elements C, O, and N in the catalyst were found to be 60.06%, 27.18%, and 12.03%, respectively.

**Fig. 7:**
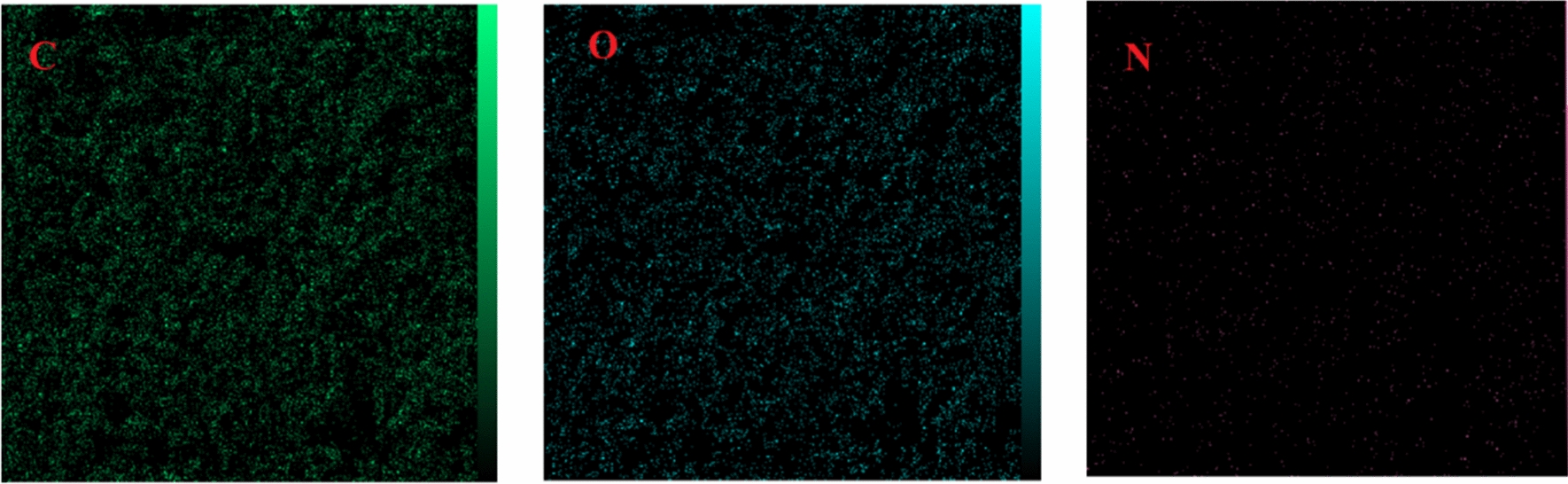
EDS-map analysis of nano-GO/3-aminopyridine

EDS-map analysis of the catalyst (Fig. 7) shows the dispersion of the elements uniformly. As a result, the organic compound is not clumped on the substrate.

#### XRD pattern of the composite

**Fig. 8:**
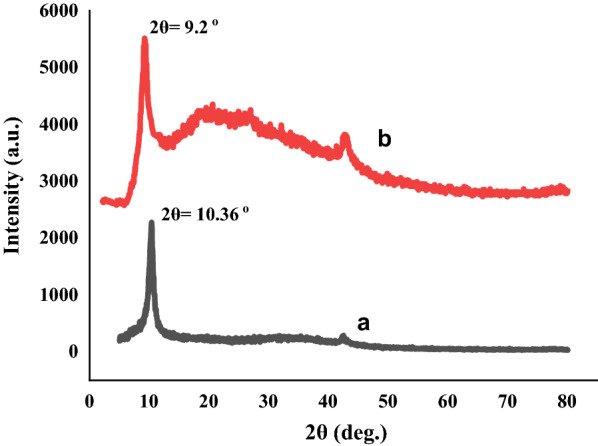
XRD pattern of GO (**a**) and nano-GO/3-aminopyridine (**b**)

Figure 8 shows the XRD patterns of GO and nano-GO/3-aminopyridine. GO and functionalized GO have index peaks in the region 2θ = 9.2°. The spacing between the plates was calculated from the Bragg equation, as follows:

$${\text{n}}\lambda \, = \,{\text{2d sin}}\theta$$

The distance between the GO plates with 2θ = 10.36° is equal to 0.85 [26] while for functionalized GO, the peak is transferred to 2θ = 9.2° and the distance between the plates is increased to 0.96. As a result, the presence of organic compounds on the GO surface increases the distance between the plates, which in turn causes the XRD peak to shift (Fig. 8).

#### TGA pattern of nano-GO/3-aminopyridine

**Fig. 9:**
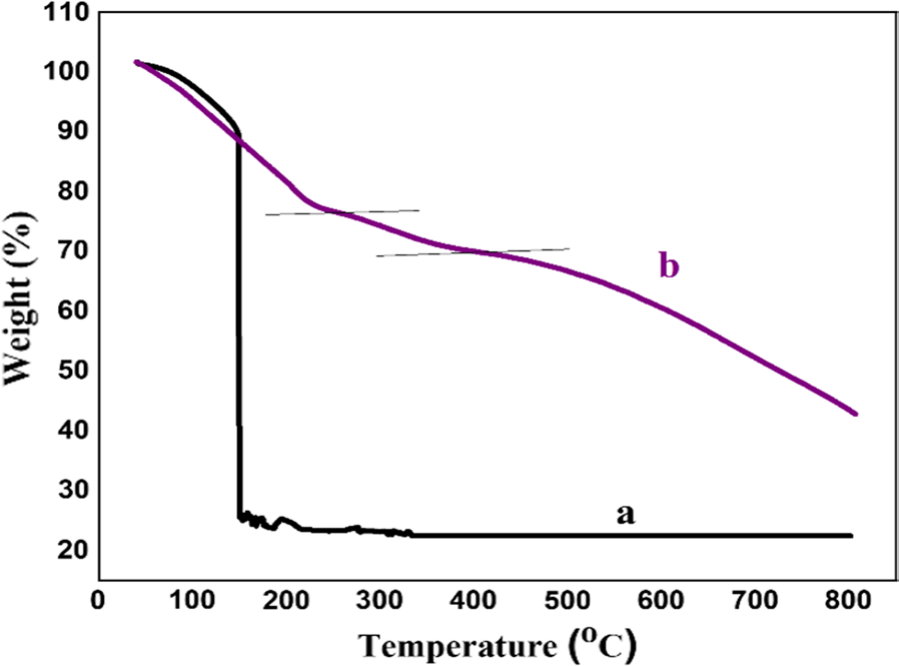
TGA pattern of GO (**a**) and nano-GO/3-aminopyridine (**b**)

The TGA curves of GO [26] and functionalized GO with 3-aminopyridine were shown in Fig. 9. Based on TGA, weight loss is observed in three stages. Initially, weight loss (about 5%) at a temperature of about 100 °C is related to the removal of adsorbed moisture from the catalyst structure. A rapid mass reduction at 200 °C and a mass reduction between 230 °C-400 °C are related to the removal of hydroxy groups from the GO surface. Finally, the mass loss at temperatures above 560 °C is related to the complete decomposition of GO. According to the results, it can be said that the placement of organic groups on the surface of GO has caused its greater thermal gravimetric analysis (Fig. 9).

#### The catalytic activity of the catalyst

**Fig. 10:**
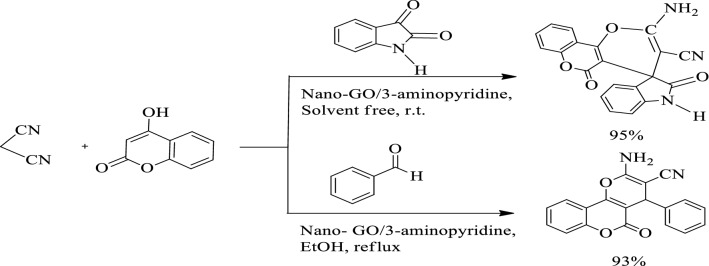
Synthesis of spiro-indoline-pyranochromene and dihydropyranochromene derivatives in the presence of nano-GO/3-aminopyridine

The efficiency of the catalyst to perform various chemical reactions is considered as one of the advantages of the catalyst. Here also the catalytic activity of the new catalyst was investigated in the synthesis of spiro-indoline-pyranochromene and dihydropyranochromene derivatives (Fig. 10). Although our new catalyst was effective in both of the above reactions, but in this study only the synthesis of spiro-indoline-pyranochromene derivatives was followed by optimization and synthesis of different derivatives.

#### Optimization of the reaction conditions

To optimize the reaction conditions, different parameters such as solvent, temperature and amount of the catalyst were investigated. In this regard, the three-component reaction of malononitrile, 4-hydroxycoumarin, and isatin was selected as a model reaction. In the first step to examine the catalytic properties of the catalyst, the reaction was investigated in ethanol–water and room temperature in the presence of GO, 3-aminopyridine and nano-GO/3-aminopyridine. The results emphasized the suitability of 3-aminopyridine and GO/3-aminopyridine as catalysts. Carrying out this reaction without the presence of a catalyst confirms the significant effect of the catalyst in advancing the reaction (Table 1, Rows 1–4).

**Table 1:** Optimization of the reaction conditions for synthesis of spiro-indoline-pyranochromene derivatives^a^

Entry	Catalyst	Solvent	Temp. (°C)	Time (min)	Weight catalyst(g)	Yield (%)
1	–	EtOH:H_2_O (1:1)	r.t	60	–	40
2	GO	EtOH:H_2_O (1:1)	r.t	40	0.04	56
3	3-aminopyridine	EtOH:H_2_O (1:1)	r.t	40	0.04	87
4	Nano-GO/3-aminopyridine	EtOH:H_2_O (1:1)	r.t	30	0.04	85
5	Nano-GO/3-aminopyridine	H_2_O	r.t	40	0.04	70
6	Nano-GO/3-aminopyridine	EtOH	r.t	15	0.04	90
**7**	**Nano-GO/3-aminopyridine**	**Solvent-free**	**r.t**	**20**	**0.04**	**95**
8	Nano-GO/3-aminopyridine	Solvent-free	70	20	0.04	95
9	Nano-GO/3-aminopyridine	Solvent-free	r.t	20	0.05	95
10	Nano-GO/3-aminopyridine	Solvent-free	r.t	37	0.03	80
11	Nano-GO/3-aminopyridine	Solvent-free	r.t	45	0.02	70

^a^1.0 mmol of malononitrile, 4-hydroxycoumarin and isatin

In the next step, the reaction was performed in different solvents and also solvent-free condition. The best result was obtained when the model reaction was performed under solvent-free condition (Table 1, Rows 4–7). Then, to find the optimal temperature, the model reaction was investigated at both room temperature and 70 °C. Increasing the temperature did not affect the reaction progress and the room temperature was selected due to its economy (Table 1, Rows 7–8). To evaluate the best amount of catalyst, the reaction was performed in different amounts of catalyst. 0.04 mg of catalyst was selected as the optimize value (Table 1, Rows 7, 9–12). So room temperature, solvent-free and 0.04 g of catalyst (Raw 7- the bold values) were selected as the optimize conditions.

#### Synthesis of spiro-indoline-pyranochromene derivatives under optimized conditions

After finding the best conditions for the model reaction, spiro-indoline-pyranochromene derivatives were synthesized under these conditions (Table 2).

**Table 2:** Synthesis of spiro-indoline-pyranochromene derivatives^a^

Entry	Products	Chemical formula	M.P. (°C)	Yields (%)
*4a*		C_20_H_11_N_3_O_4_	290–293 (27)	95
*4b*		C_25_H_22_N_2_O_6_	208–212(19)	88
*4c*		C_26_H_22_N_2_O_6_	224–227(19)	88
*4d*		C_26_H_22_N_2_O_6_	257–258(19)	92
*4e*		C_28_H_20_N_2_O_6_	280–281(19)	93
*4f*		C_26_H_24_N_2_O_6_	218–220 (19)	87
*4 g*		C_27_H_24_N_2_O_6_	228–230 (19)	87
*4 h*		C_29_H_22_N_2_O_6_	233–235 (19)	90

^a^Reaction conditions: 1.0 mmol of isatin ring, nitrile moiety, 4-hydroxy coumarin and 0.04 g catalyst under solvent-free at r.t

#### Proposed mechanism of spiro-indoline-pyranochromene derivatives

**Fig. 11:**
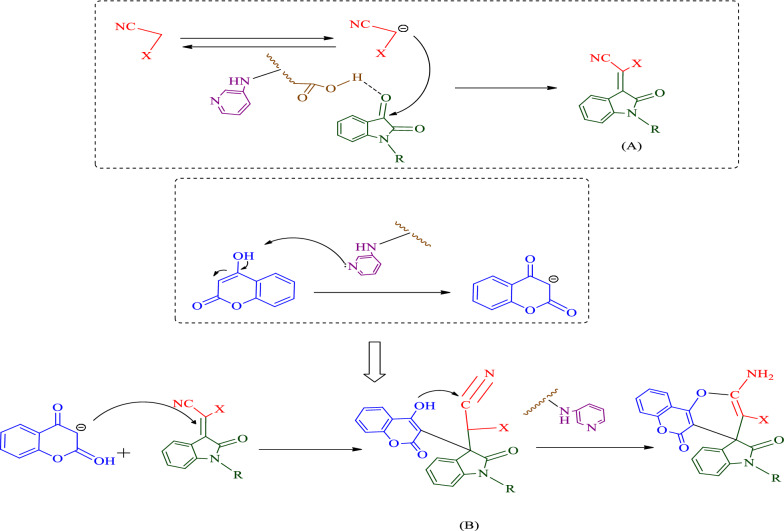
Proposed mechanism for synthesis of spiro-indoline-pyranochromene derivatives

Initially, acidic sites in the catalyst interact with isatin oxygen group. The carboxylic acid groups of GO can react to the carbonyl groups of starting materials and play an acidic role. On the other hand, nitrogen groups of 3-aminopyridine can take the acidic hydrogens of the starting materials and play a base role. Then malononitrile which is activated by basic sites of the catalyst reacts with the carbonyl group of isatin and form the Knoevenagel product (A). 4-Hydroxycoumarin then forms intermediate B according to Michael's addition reaction. Finally, by intramolecular reaction and closing of the product ring, the catalyst is removed from the reaction cycle (Fig. 11).

#### Reusability of the catalyst

**Fig. 12:**
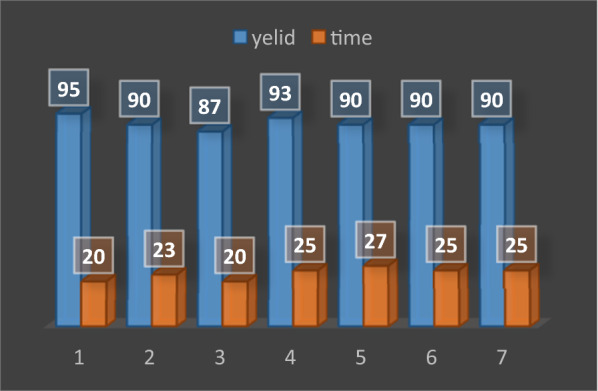
Reusability of the catalyst

**Fig. 13:**
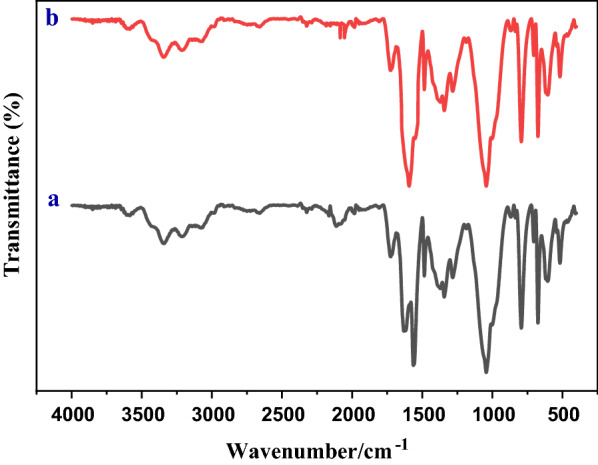
FT-IR spectrum of the nano-GO/3-aminopyridine before (**a**) and after the recycling (**b**)

To evaluate the reusability of the catalyst, nano-GO/3-aminopyridine isolated from the reaction medium was washed with hot ethanol and then dried at 60 °C overnight. The dried catalyst was used in subsequent iterations of the model reaction, the results of which were recorded in Fig. 12. As indicated in this figure the catalyst was effective up to 7 runs without any significant decrease in its performance. FT-IR spectra of the catalyst before and after the recycling also showed in Fig. 13. In these spectra no remarkable changes were apperceived.

#### Hot filtration test

Hot filtration test also was done on the model reaction to demonstrate the heterogeneous nature and the recoverability of the nano-GO/3-aminopyridine. 1.0 mmol of isatin, malononitrile and 4-hydroxy coumarin were mixed at room temperature in the presence of nano-GO/3-aminopyridine (0.04 g) for 10 min. Then, the catalyst was discrete from the mixture by filtration and the reaction was monitored. However, no improvement in promote of reaction was observed in the filtrate.

#### Comparison of the efficiency of nano-GO/3-aminopyridine with the other reported catalysts

To demonstrate the advantages of the reported method for the synthesis of spiro-indoline-pyranochromene derivatives, we compared the catalytic activity of nano-GO/3-aminopyridine with the other catalysts reported in the previous works (Table 3). According to the results, nano-GO/3-aminopyridine can be reported as an efficient catalyst in terms of time and efficiency compared to the other reported catalysts. These promising results should be attributed to the cooperation of acidic and alkaline groups in the catalyst. In addition, the homogeneous and suitable distribution of 3-aminopyridine over a large surface area of graphene has resulted in better catalyst performance. Using of toxic solvent (CH_3_CN), homogeneous catalyst and the length of the reaction time are the weaknesses of the other reports [28]. The results showed that our method is preferred in comparison to the other works.

**Table 3:** Comparison of the nano-GO/3-aminopyridine with other catalysts in the synthesis of spiro-indoline-pyranochromene derivatives

Entry	Catalyst/Temp/Time/Solvent	Yield (%)	Refs.
1	[Dabco-H]Cl /50^O^C /2 h /CH_3_CN	95	[28]
2	BN@Fe_3_O_4_ /80 °C/45 min/Water	88	[29]
3	Et_3_N/75^O^C /45 min /EtOH	90	[19]
4	Nano-GO/3-aminopyridine/r.t/20 min/solvent-free	**95**	**This work**

## Conclusion

We presented the synthesis and identification of a new acid–base dual-use catalyst. The catalyst was synthesized simply and cleanly in ethanol solvent, at reflux temperature without the need for any organic, toxic or volatile substances. Small molecules of 3-aminopyridine were stabilized on the surface of nano-layers GO by covalent bonds. Catalyst structure was investigated using FT-IR, FESEM, EDX, and map analyses. Stabilization of 3-aminopyridine molecules on the surface of graphene caused porosity, increased sheet spacing and layered structure of GO which were effective in improving catalyst efficiency. The ability of the catalyst was investigated in synthesis of two categories of compounds. The synthesis of spiro-indoline-pyranochromene derivatives was successfully performed in the presence of the catalyst under solvent-free conditions with high efficiency. Reaction under mild conditions, short time, good efficiency and easy purification, cheap and non-toxic nature of the catalyst, use of a new catalytic system and reusability of the catalyst were the notable advantages of this method. Results revealed that bonding of organic molecules by covalent bonding on the surface of GO increased the catalyst recoverability.

## Supplementary Information

**Additional file 1.** Spectroscopic data for the synthesized Spiro-indoline-pyranochromene derivative.

## Data Availability

All data generated or analyzed during this study are included in this published article.

## Abbreviations

GO: Graphene oxide
FESEM: Field emission scanning electron microscope
XRD: X-ray diffraction
EDX: Energy-dispersive X-ray
TGA: Thermos gravimetric analysis
EDS: Dispersive X-ray spectroscopy
TLC: Thin layer chromatography
EtOH: Ethanol
