# Supplementary material for: A novel heterogeneous acid–base nano-catalyst designed based on graphene oxide for synthesis of spiro-indoline-pyranochromene derivatives
Source: BMC Chem. 2023 Mar 10;17(1):12. doi: 10.1186/s13065-023-00930-5 (PMC9999615; doi:10.1186/s13065-023-00930-5)
Supplement: Supplementary file 1 — Additional file 1. Spectroscopic data for the synthesized Spiro-indoline-pyranochromene derivative. [file 13065_2023_930_MOESM1_ESM.docx]

**A Novel Heterogeneous acid-base nano-Catalyst Designed Based on Graphene Oxide for Synthesis of Spiro-indoline-pyranochromene derivatives**

**Soghra Khabnadideh , Khashayar Khorshidi , Leila Amiri-Zirtol ^[[1]](#footnote-1)^**

^1^Pharmaceutical Science Research Center, Shiraz University of Medical Sciences, Shiraz, Iran.

Author contributions: Soghra Khabnadideh : Conceptualization, Writing- Reviewing and Editing. Khashayar Khorshidi : expremental. Leila Amiri-Zirtol: Conceptualization, Investigation, Writing- Original. Preparing the manuscript was done by all authors.


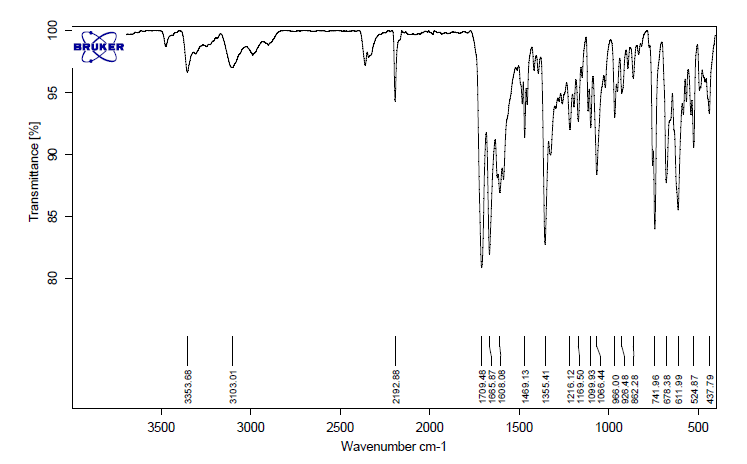


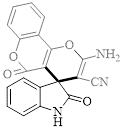


The IR spectrum of *2'*-amino-*2,5'*-dioxo-*5'H*-spiro [indoline-*3,4*'-pyrano[*3,2*-*c*]chromene]-3'-carbonitrile (4a)


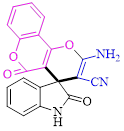

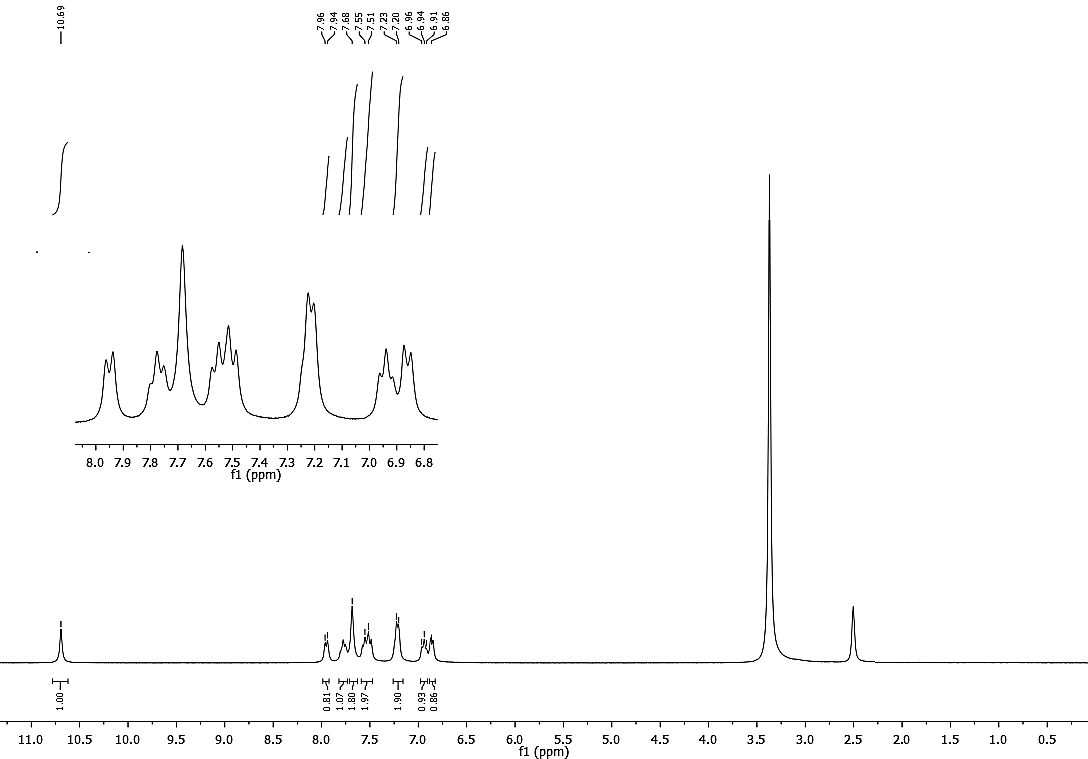


The ^1^H-NMR spectrum of *2'*-amino-*2,5'*-dioxo-*5'H*-spiro [indoline-3,4'-pyrano[*3,2-c*]chromene]-3'-carbonitrile (4a)


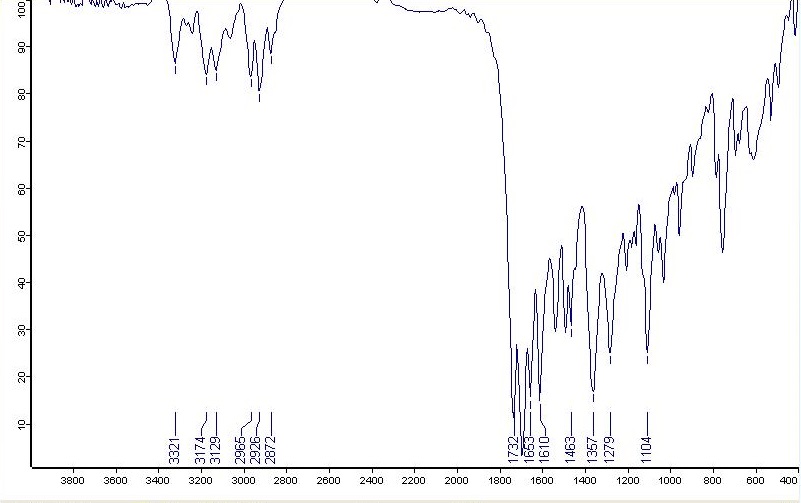

The IR (KBr cm^-1^) spectrum of methyl *2'*-amino-1-butyl-2,5'-dioxo-*5'H*-spiro[indoline-*3,4'*-pyrano[*3,2-c*] chromene]-3'-carboxylate (4b)


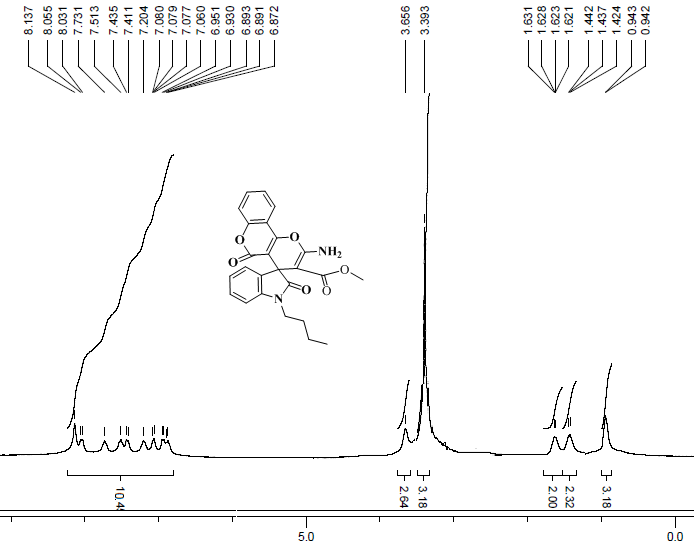


The ^1^H-NMR spectrum of methyl *2'*-amino-1-butyl-*2,5'*-dioxo-*5'H*-spiro[indoline-*3,4'*-pyrano[*3,2-c*] chromene]-3'-carboxylate (4b)


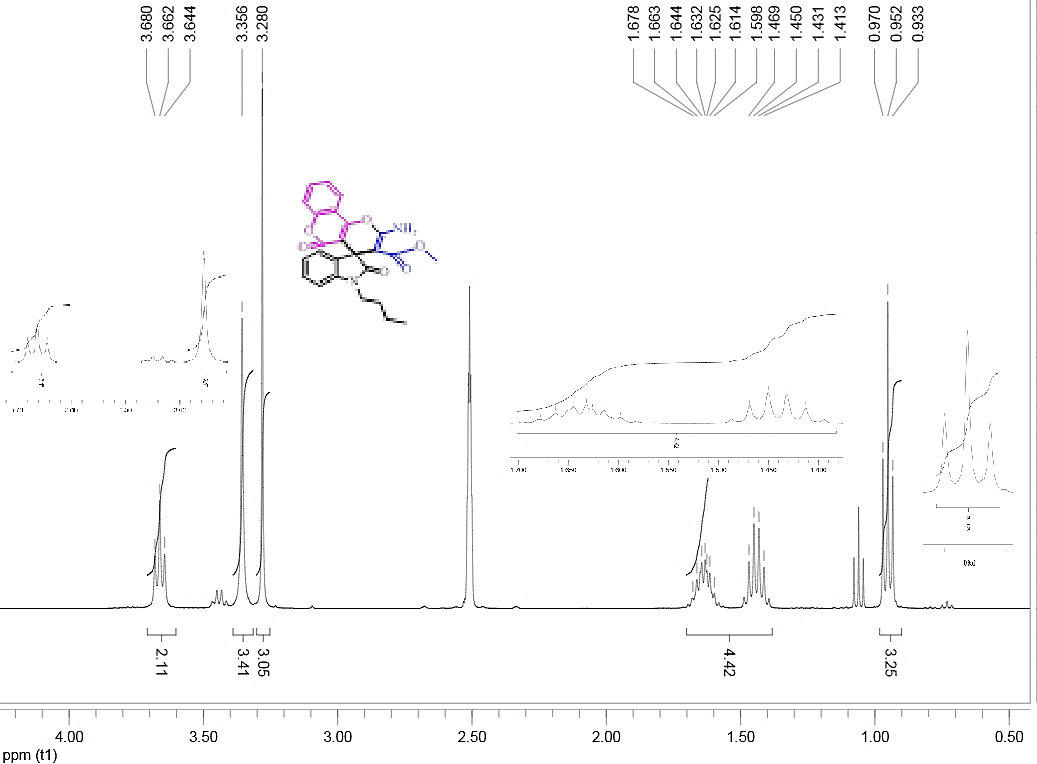


The^1^H-NMR spectrum of methyl *2'*-amino-1-butyl-*2,5'*-dioxo-*5'H*-spiro[indoline-*3,4'*-pyrano[*3,2-c*]chromene]-3'-carboxylate (4b)


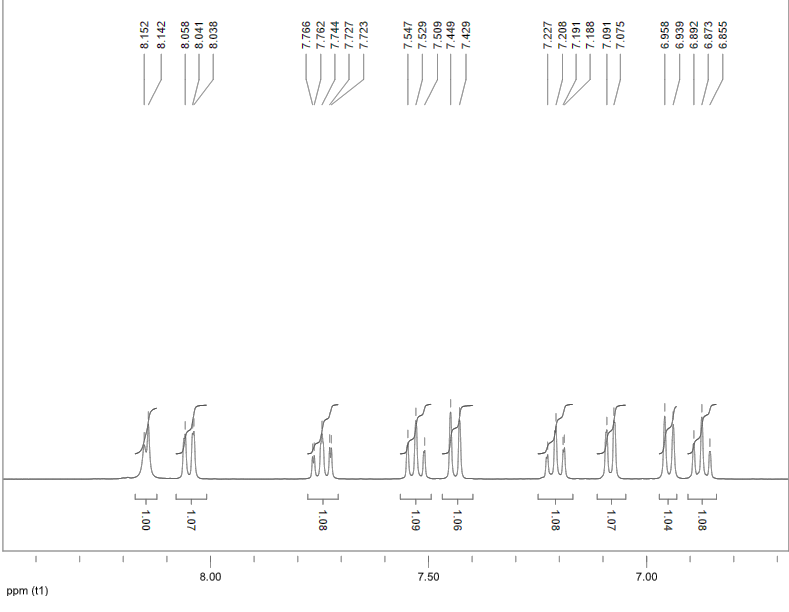

The ^1^H-NMR spectrum of methyl *2*'-amino-1-butyl-*2,5'*-dioxo-*5'H*-spiro[indoline-3,4'-pyrano[*3,2-c*]chromene]-3'-carboxylate (4b)


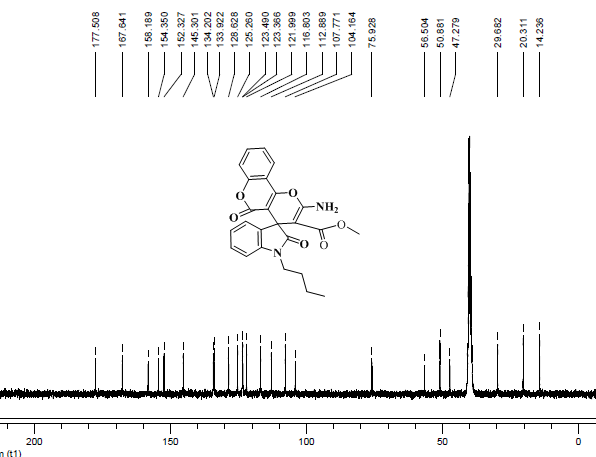


The ^13^C-NMR spectrum of methyl *2'*-amino-1-butyl-*2,5'*-dioxo-*5'H*-spiro[indoline-*3,4'*-pyrano[*3,2-c*] chromene]-*3'*-carboxylate (4b)


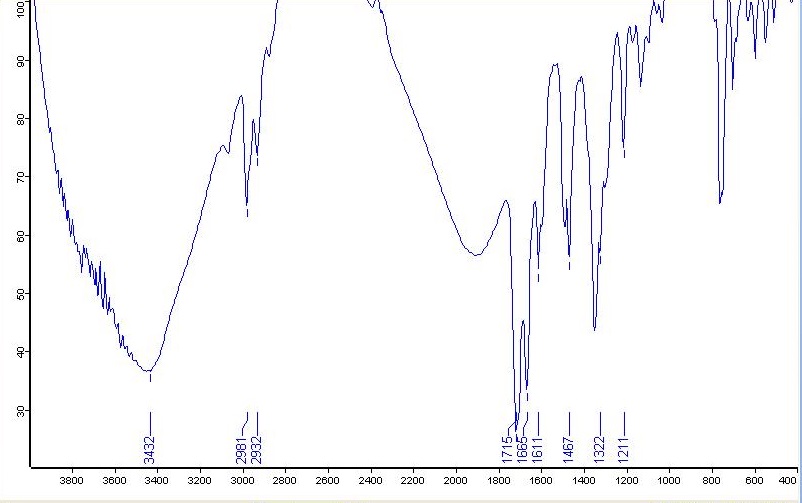

The IR spectrum of methyl *2'*-amino-1-(3-methylbut-2-en-1-yl)-*2,5'*-dioxo-*5'H*-spiro[indoline-3,4'-pyrano[*3,2-c*] chromene]-3'-carboxylate (4c)


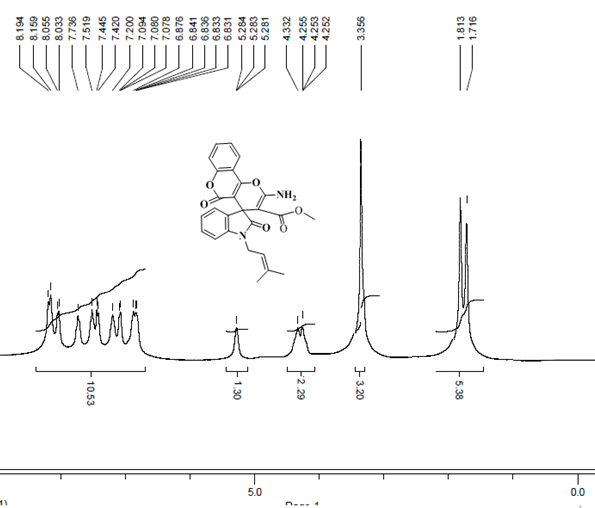


The ^1^H-NMR spectrum of methyl *2*'-amino-1-(3-methylbut-2-en-1-yl)-2,5'-dioxo-*5'H*-spiro[indoline-*3,4'*-pyrano[*3,2-c*] chromene]-3'-carboxylate (4c)


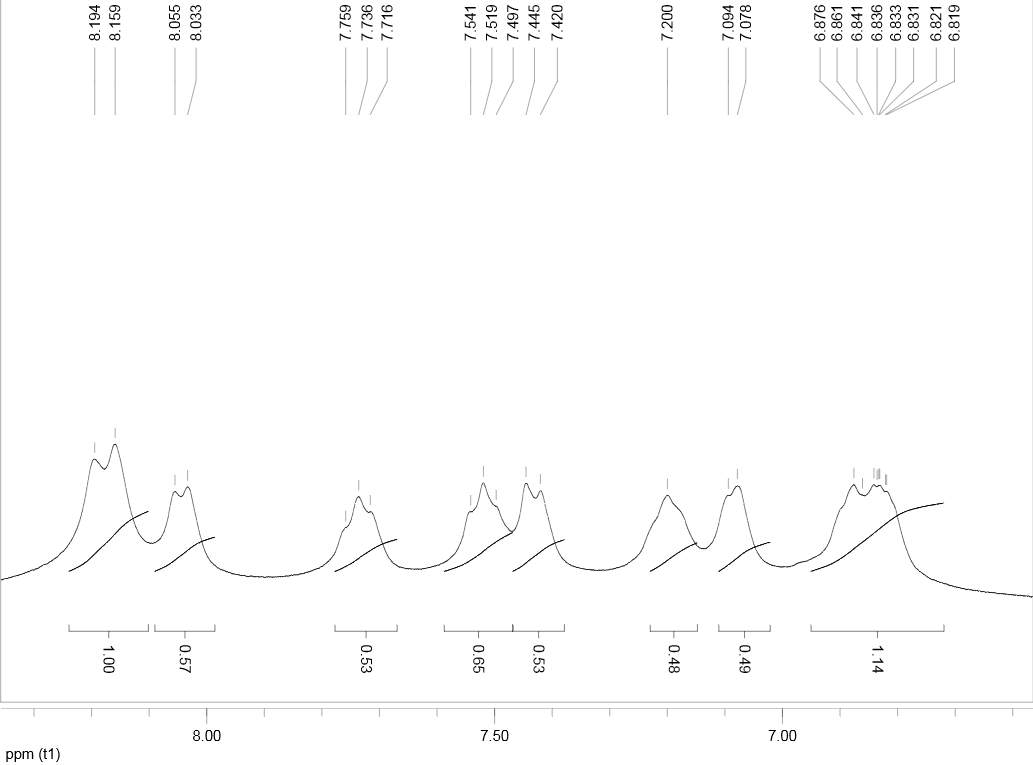

The ^1^H-NMR spectrum of methyl *2*'-amino-1-(3-methylbut-2-en-1-yl)-*2,5'*-dioxo-*5'H*-spiro[indoline-*3,4'*-pyrano[*3,2-c*]chromene]-3'-carboxylate (4c)


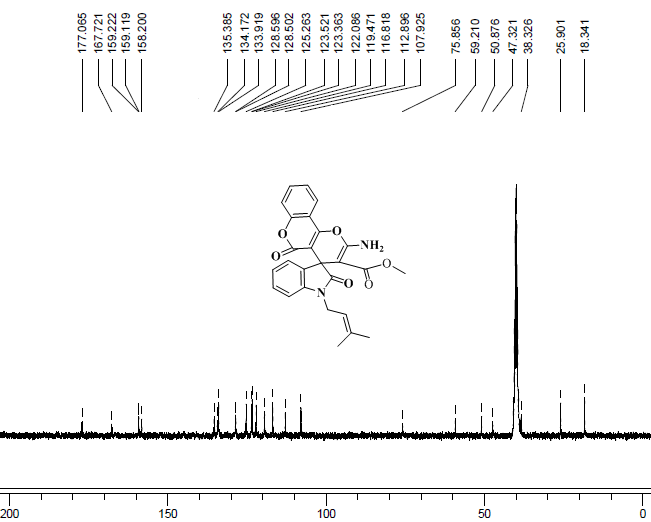


The ^13^C-NMR spectrum of methyl *2'*-amino-1-(3-methylbut-2-en-1-yl)-*2,5'*-dioxo-*5'H*-spiro[indoline-3,4'-pyrano[*3,2-c*] chromene]-3'-carboxylate (4c)


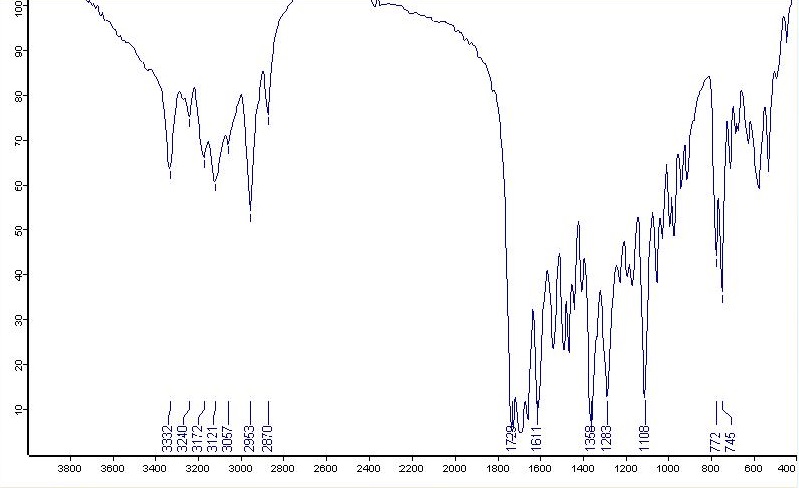

The IR spectrum of methyl *2*'-amino-1-cyclopentyl-2,5'-dioxo-*5'H*-spiro[indoline-3,4'-pyrano[*3,2-c*] chromene]-*3*'-carboxylate (4d)


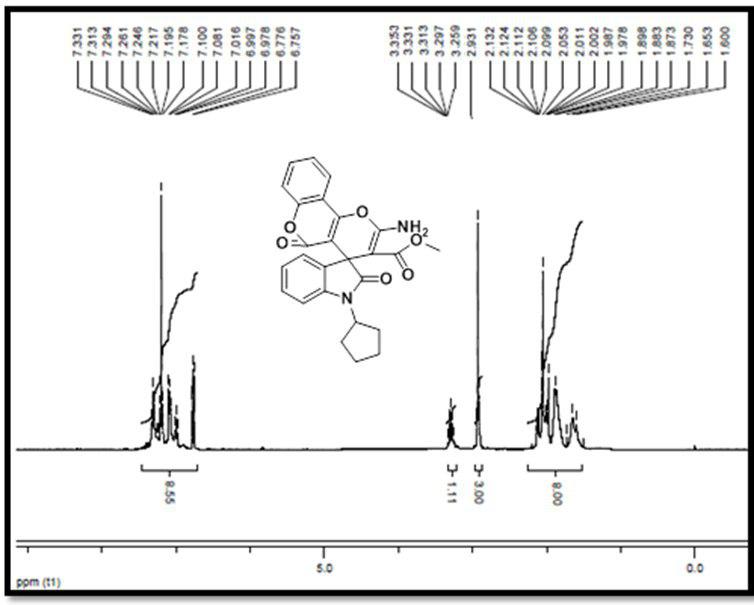


The ^1^H-NMR spectrum of methyl *2*'-amino-1-cyclopentyl-*2,5'*-dioxo-*5'H*-spiro[indoline-*3,4'*-pyrano[*3,2-c*] chromene]-3'-carboxylate (4d)


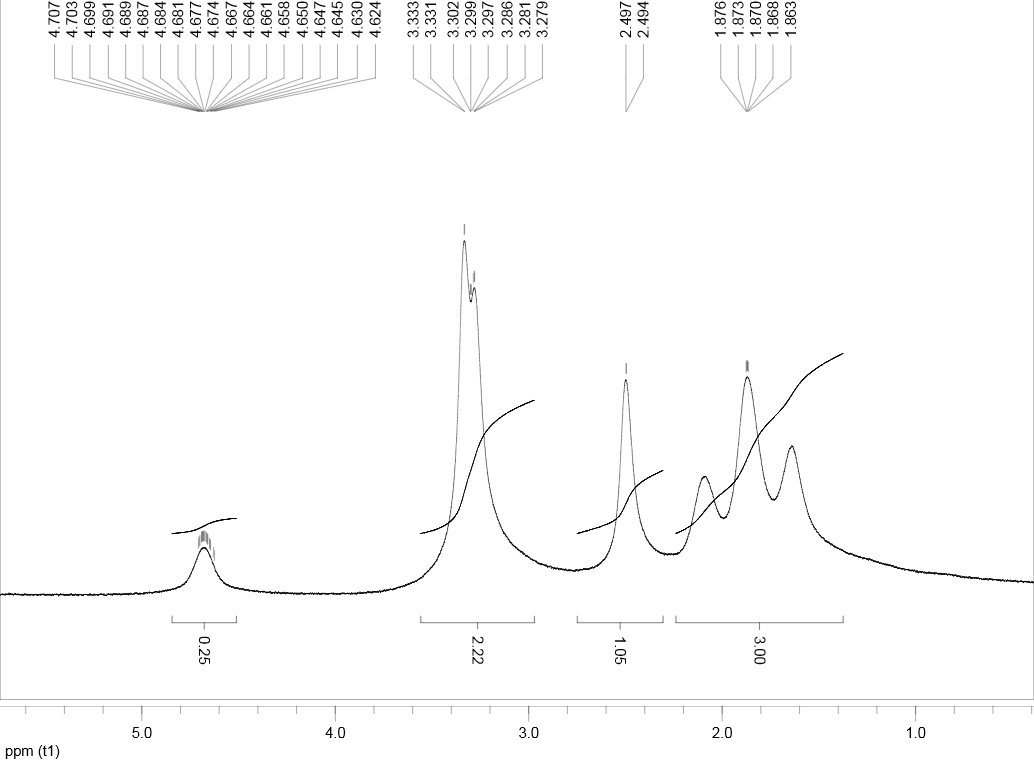


The ^1^H-NMR spectrum of methyl *2*'-amino-1-cyclopentyl-*2,5'*-dioxo-*5'H*-spiro[indoline-*3,4'*-pyrano[*3,2-c*] chromene]-3'-carboxylate (4d)


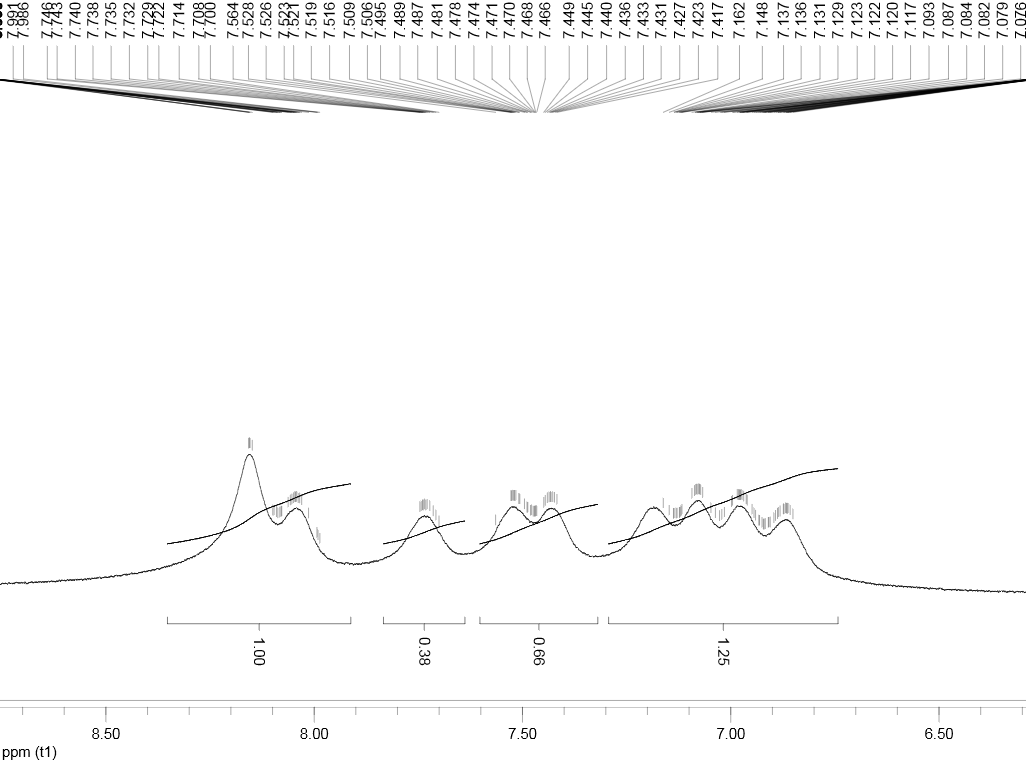


The ^1^H-NMR spectrum of methyl *2*'-amino-1-cyclopentyl-*2,5'*-dioxo-*5'H*-spiro[indoline-3,4'-pyrano[*3,2-c*] chromene]-3'-carboxylate (4d)


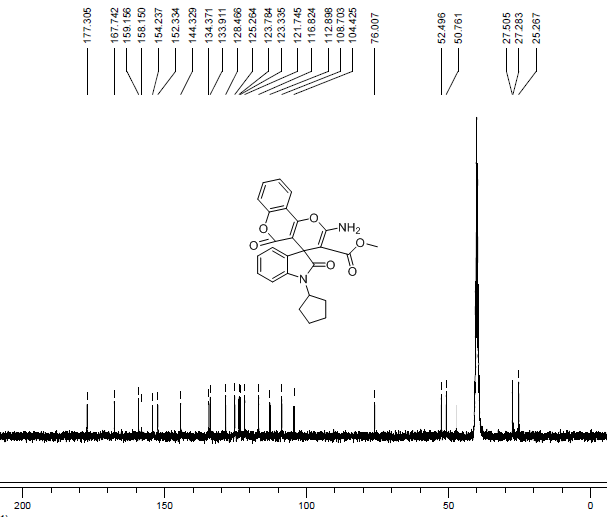


The ^13^C-NMR spectrum of methyl *2*'-amino-1-cyclopentyl-2,5'-dioxo-*5'H*-spiro[indoline-3,4'-pyrano[*3,2-c*] chromene]-3'-carboxylate (4d)


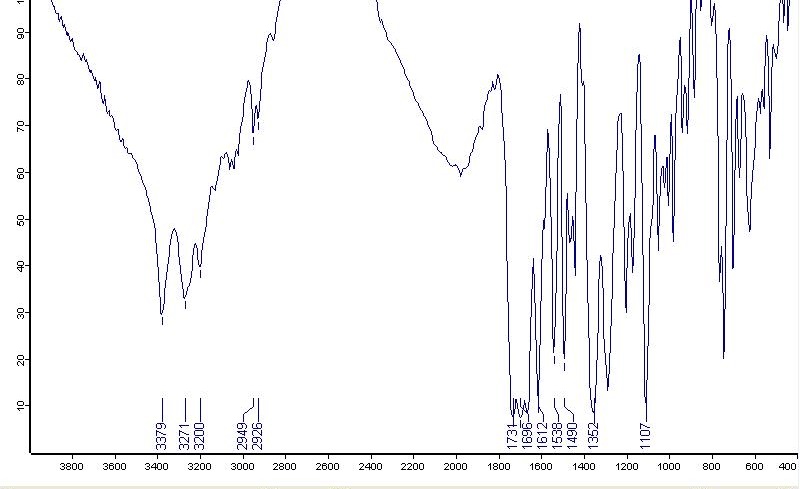

The IR spectrum of methyl *2*'-amino-1-benzyl-*2,5'*-dioxo-*5'H*-spiro[indoline-*3,4'*-pyrano[*3,2-c*] chromene]-3'-carboxylate (4e)


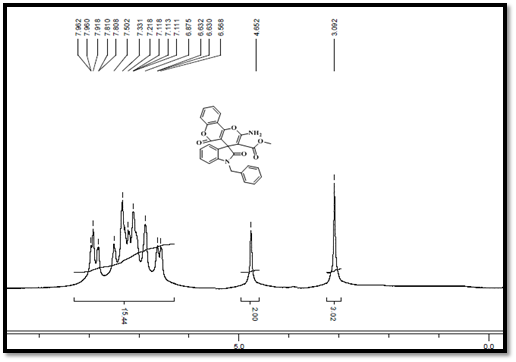


The ^1^H-NMR spectrum of methyl *2*'-amino-1-benzyl-*2,5'*-dioxo-*5'H*-spiro[indoline-*3,4'*-pyrano[*3,2-c*] chromene]-3'-carboxylate (4e)


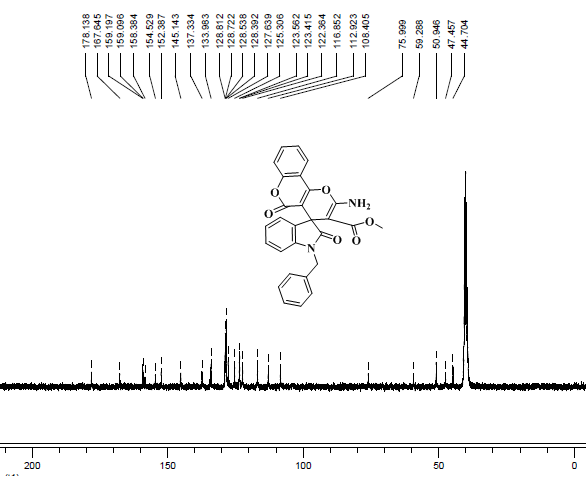


The ^13^C-NMR spectrum of methyl *2*'-amino-1-benzyl-*2,5'*-dioxo-*5'H*-spiro[indoline-3,4'-pyrano[*3,2-c*] chromene]-3'-carboxylate (4e)


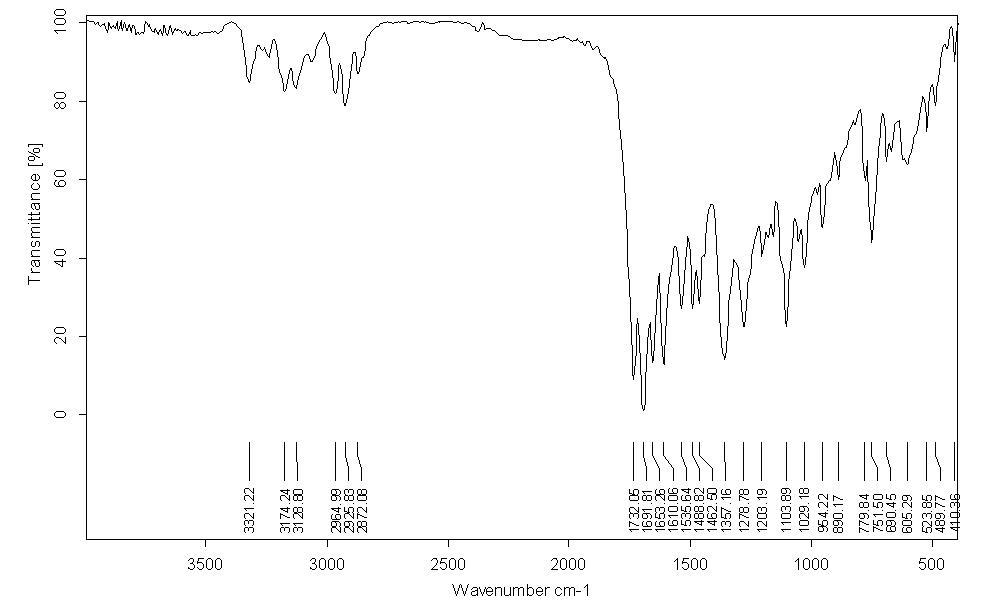

The IR spectrum of ethyl *2*'-amino-1-butyl-*2,5'*-dioxo-5'H-spiro[indoline-*3,4'*-pyrano[*3,2-c*] chromene]-*3'*-carboxylate (4f)


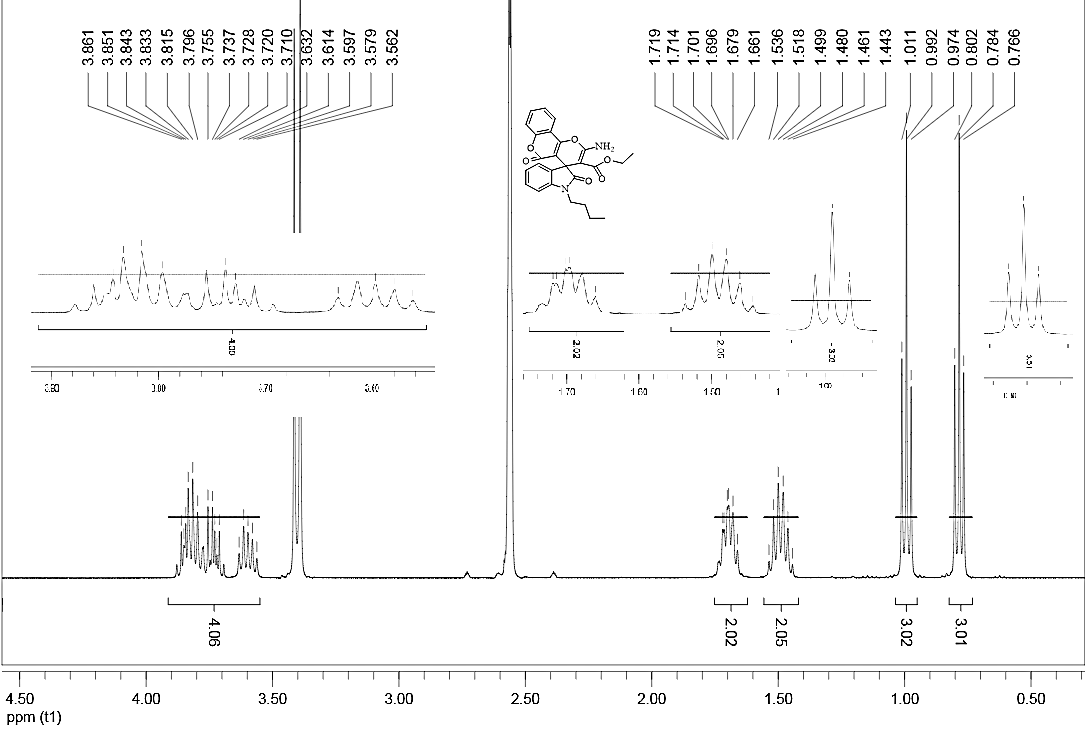


The ^1^H-NMR spectrum of ethyl *2'*-amino-1-butyl-*2,5'*-dioxo-*5'H*-spiro[indoline-*3,4'*-pyrano[*3,2-c*] chromene]-3'-carboxylate (4f)


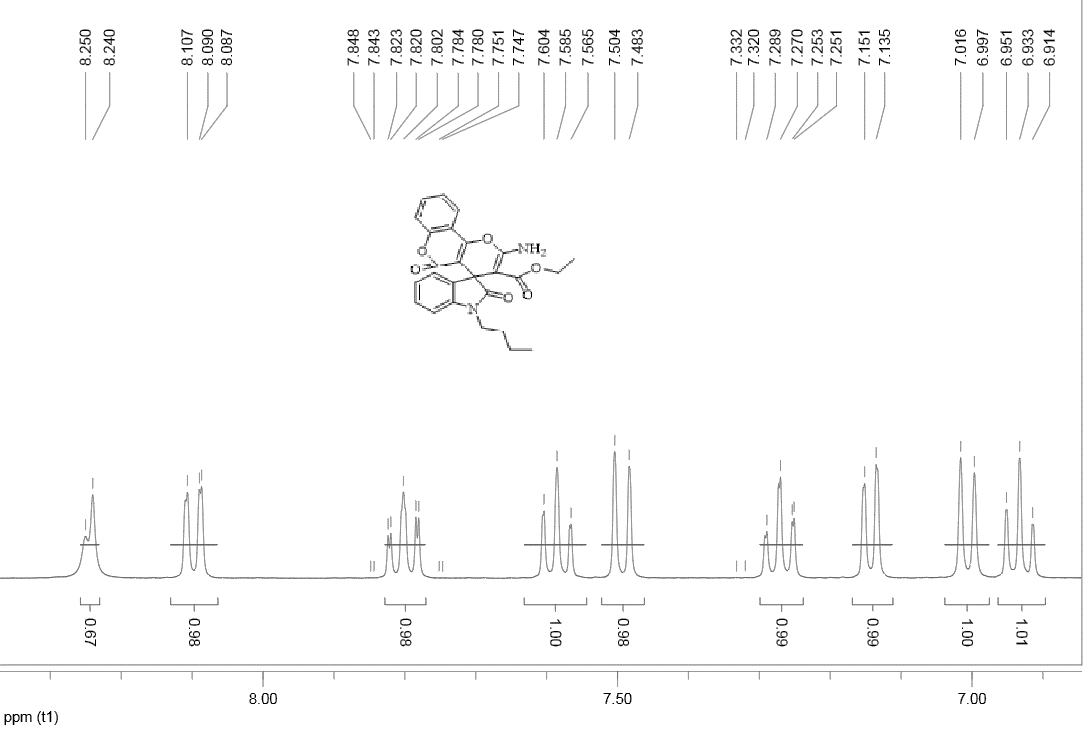


The ^1^H-NMR spectrum of ethyl *2'*-amino-1-butyl-*2,5'*-dioxo-*5'H*-spiro[indoline-*3,4'*-pyrano[*3,2-c*] chromene]-*3'*-carboxylate (4f)


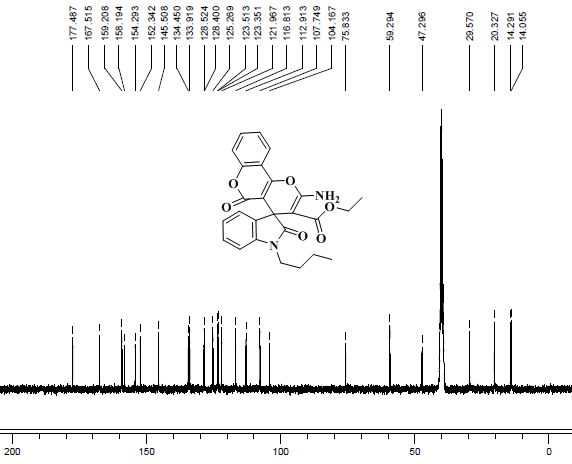


The ^13^C-NMR spectrum of ethyl *2'*-amino-1-butyl-*2,5'*-dioxo-*5'H*-spiro[indoline*-3,4'-*pyrano[*3,2-c*] chromene]-3'-carboxylate (4f)


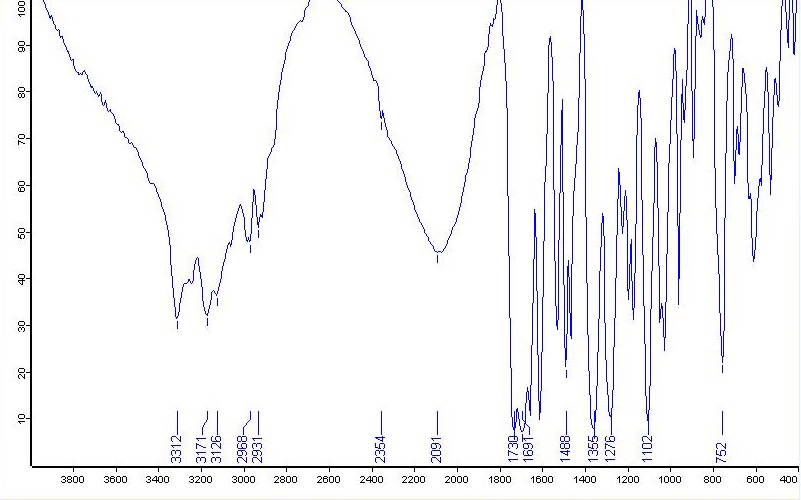

The IR spectrum of ethyl *2*'-amino-1-(3-methylbut-2-en-1-yl)-*2,5'*-dioxo-*5'H*-spiro[indoline-*3,4'*-pyrano[*3,2-c*] chromene]-3'-carboxylate (4g)


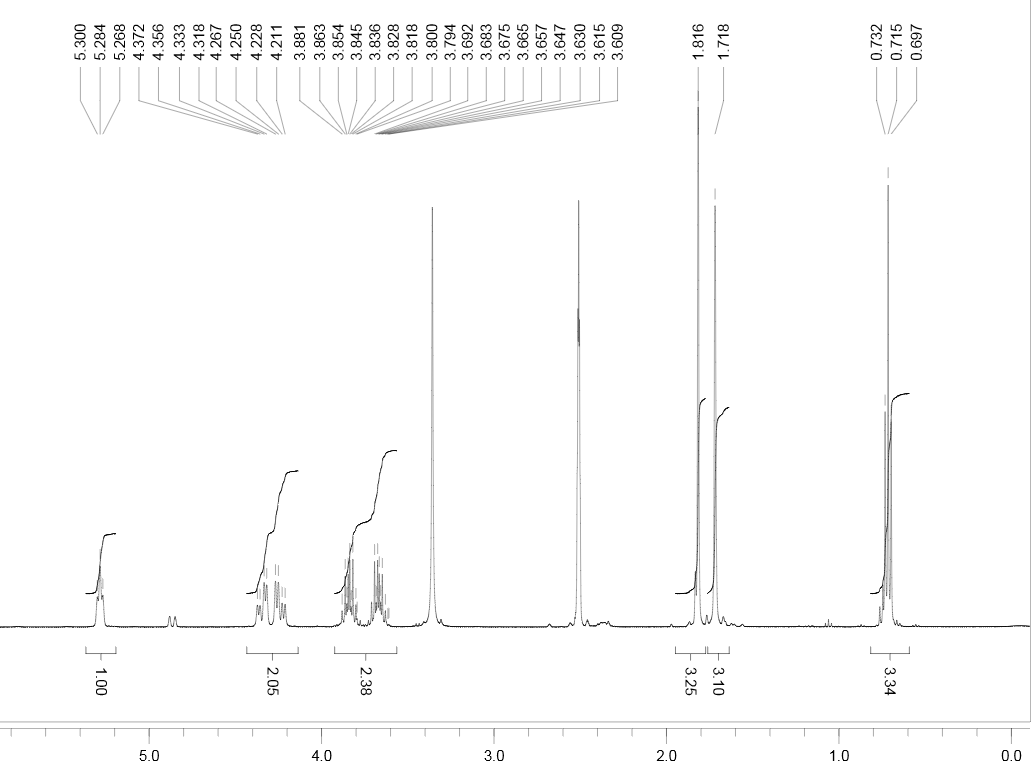


The ^1^H-NMR spectrum of ethyl *2*'-amino-1-(3-methylbut-2-en-1-yl)-*2,5'*-dioxo-*5'H*-spiro[indoline-*3,4'*-pyrano[*3,2*-*c*] chromene]-3'-carboxylate (4g)


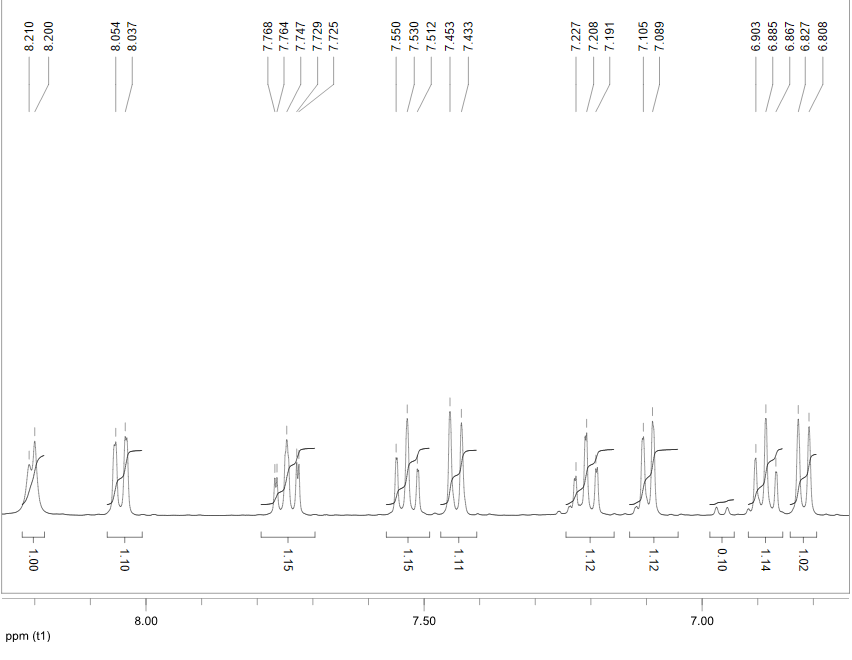

The ^1^H-NMR spectrum of ethyl *2'*-amino-1-(3-methylbut-2-en-1-yl)-*2,5'*-dioxo-*5'H*-spiro[indoline-*3,4'*-pyrano[*3,2-c*] chromene]-3'-carboxylate (4g)


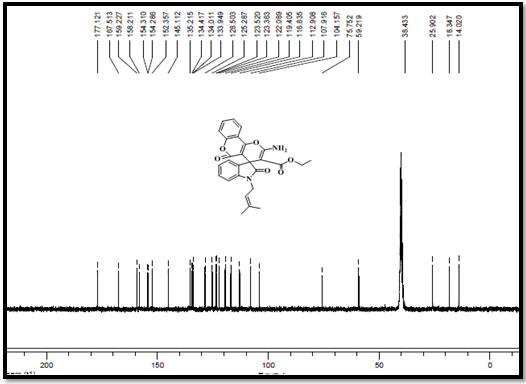


The ^13^C-NMR spectrum of ethyl *2*'-amino-1-(3-methylbut-2-en-1-yl)-*2,5'*-dioxo-*5'H*-spiro[indoline-3,4'-pyrano[*3,2-c*] chromene]-*3*'-carboxylate (4g)


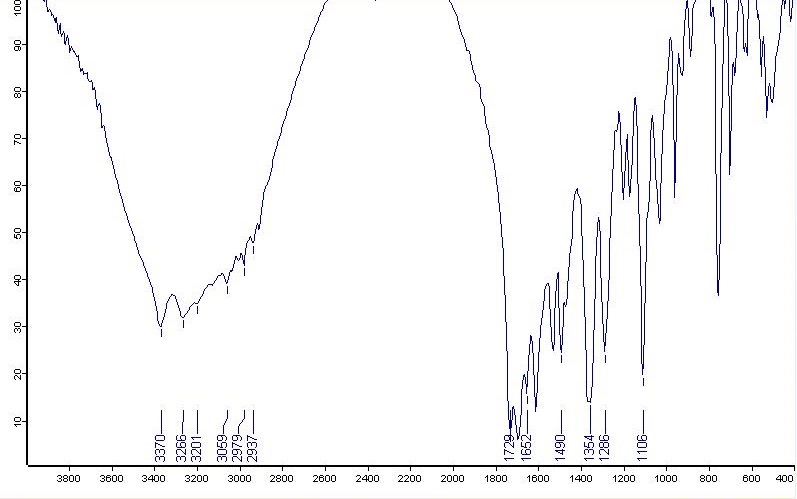

The IR spectrum of ethyl 2'-amino-1-benzyl-2,5'-dioxo-5'H-spiro[indoline-3,4'-pyrano[*3,2-c*] chromene]-3'-carboxylate (4h)


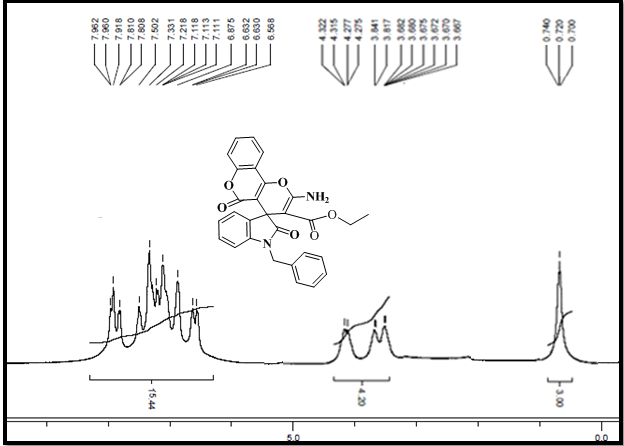


The ^1^H-NMR spectrum of ethyl *2'*-amino-1-benzyl-*2,5'*-dioxo-*5'H*-spiro[indoline-3,4'-pyrano[*3,2-c*] chromene]-3'-carboxylate (4h)


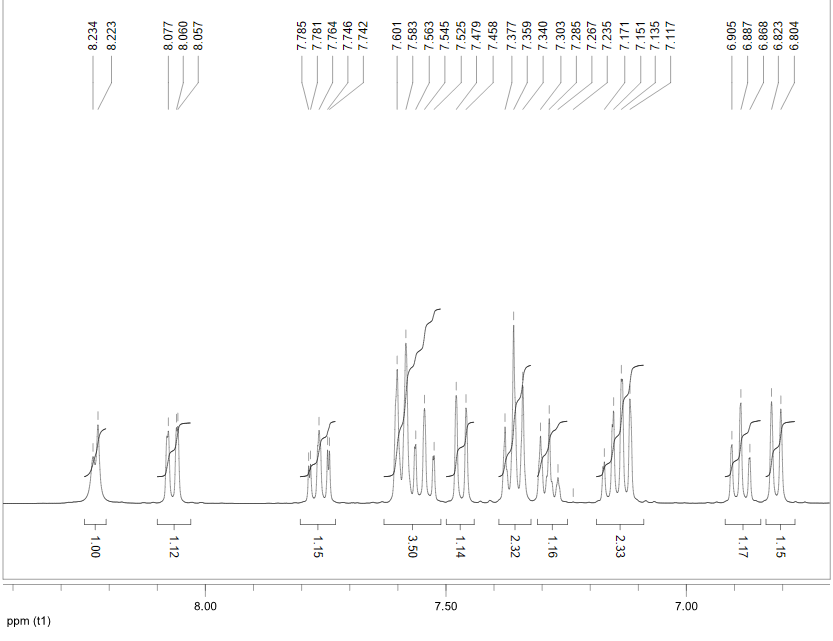

The ^1^H-NMR spectrum of ethyl *2'*-amino-1-benzyl-*2,5*'-dioxo-*5'H*-spiro[indoline-*3,4'*-pyrano[*3,2-c*] chromene]-3'-carboxylate (4h)


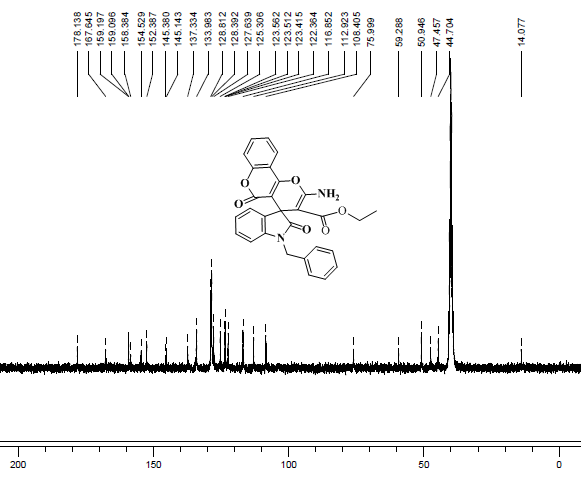


The ^13^C-NMR spectrum of ethyl 2'-amino-1-benzyl-2,5'-dioxo-5'H-spiro[indoline-*3,4'-*pyrano[*3,2-c*] chromene]-3'-carboxylate (4h)

1. Corresponding author: Leila Amiri-Zirtol ,Tel.: +98-71-32424127-8; Fax: +98-71-32424126; E-mail: [l.amiri@rocketmail.com](mailto:l.amiri@rocketmail.com)

   Pharmaceutical Science Research Center, Shiraz University of Medical Sciences, Shiraz, Iran.

   Full list of author information is available at the end of the article. [↑](#footnote-ref-1)
